# Traumatic Hemifacial Avulsion and Degloving Injury With Left Zygomaticomaxillary Complex Fracture: A Case Report

**DOI:** 10.7759/cureus.59777

**Published:** 2024-05-07

**Authors:** V Vaishnavi, Monisha J Shetty, Adarsh R Shekhar, Nandesh Shetty

**Affiliations:** 1 Department of Oral and Maxillofacial Surgery, AJ Institute of Dental Sciences, Mangalore, IND

**Keywords:** single-stage primary reconstruction, zygomatic complex fracture, hemifacial avulsion, globe avulsion, facial trauma management

## Abstract

Managing gross maxillofacial injuries poses significant challenges due to potential complications such as airway obstruction, cervical spine injuries, and damage to cranial structures. The resultant deformities from these injuries can have enduring psychological effects, which, if left unaddressed, can be devastating. This report outlines an approach for a patient with a history of a bull gore injury wherein a 49-year-old male presented to the Department of Oral and Maxillofacial Surgery, reporting an alleged animal attack. The patient had experienced avulsion of the left eye and degloving injuries affecting the lower eyelids, nose, left cheek, and upper and lower lips, along with skin over the chin, coupled with a left zygomaticomaxillary complex fracture. Subsequently, a comprehensive single-stage primary reconstruction and repair procedure was performed. Immediate single-stage reconstruction has shown success in achieving excellent functional and aesthetic outcomes. Preserving original tissue during debridement is crucial in preventing infection and minimizing flap loss.

## Introduction

Severe facial injuries can result from a range of traumatic events, including high-speed motor vehicle collisions, assaults, domestic violence, animal bites, and falls. These incidents, often involving high-velocity forces, pose significant challenges in terms of management due to their potential for both morbidity and mortality. Among these traumatic scenarios, extensive facial avulsion and degloving injuries, often associated with events like bull races, introduce complexities marked by airway compromise, profuse bleeding, soft tissue loss, and severe disfigurement. The effects extend beyond immediate physical consequences, encompassing enduring post-traumatic aesthetic changes and functional limitations, all of which can have profound psychological impacts [1,2]. In cases of bull horn injuries, distinctive features manifest, characterized by extensive tissue damage, varied injury paths, cavities, and a substantial microbial load, necessitating careful consideration of infection prevention strategies, including tetanus prophylaxis [1].

This case highlights the complex nature of injuries associated with bull races, focusing on presenting and managing a patient with a left zygomaticomaxillary complex (ZMC) fracture and globe avulsion.

## Case presentation

A 49-year-old male presented to the Department of Oral and Maxillofacial Surgery with a bull gore injury, reporting no loss of consciousness, vomiting, or seizures. Notably, the patient exhibited oral and nasal bleed without ear bleed. The medical history revealed no known comorbidities, drug allergies, or relevant family history.

**Figure 1:**
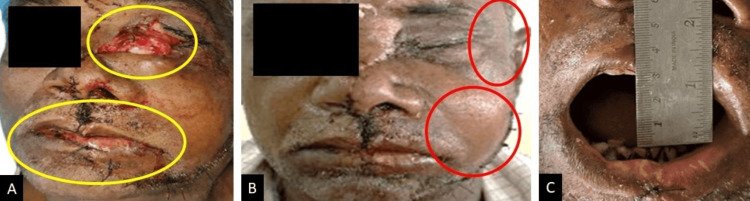
Preoperative gross features of the patient (A) Enucleation of the left eye. (B) Step and tenderness noted in relation to the left infraorbital region, flattening of the left malar region, and frontozygomatic region. (C) Restricted mouth opening of 29 mm. The regions of importance have been marked to specifically show the preoperative gross features of the patient in three different panels

Upon general examination, the patient demonstrated consciousness and orientation with a Glasgow Coma Scale (GCS) score of 15/15. Noteworthy findings included the reactivity of the right pupil to light, while the left globe exhibited rupture and avulsion. The patient displayed motor function by moving all four limbs against gravity. The ophthalmological intervention involved the enucleation of the left eye. Superficial lacerations extended from the medial canthus to the upper lip and from the midline of the lower lip along the left cheek, as shown in Figure 1.

**Figure 2:**
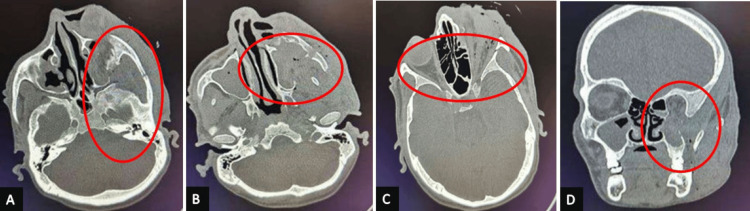
Intraoperative views on the computed tomography (A) Fracture of the left zygomatic arch, infraorbital rim, and avulsion of the left eye. (B) Fracture of the anterior and posterolateral wall of the maxillary sinus. (C) Separation of the left frontozygomatic suture. (D) Fracture of the left medial wall and floor of the orbit

**Figure 3:**
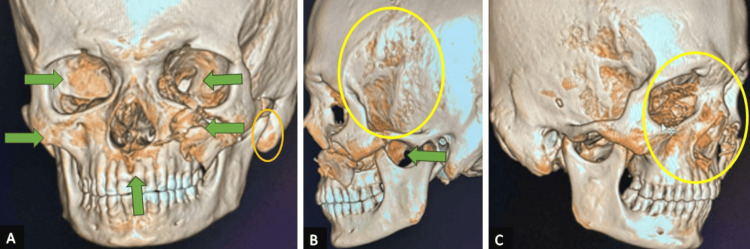
Intraoperative visualized skeletal views (A) Fracture of the left zygomatic arch. (B) Lateral displacement of the left anterior wall of the maxillary sinus. (C) Lateral and inferior displacement of the left anterior wall of the maxillary sinus with left zygomatic arch fracture. The skeletal views visualized during the surgery assisted in knowing the exact sites of lesions as marked in the panels

A computed tomography scan of axial and coronal views revealed fractures involving the left zygomatic arch, infraorbital rim, anterior and posterolateral walls of the maxillary sinus, left buttress and left medial wall, and floor of the orbit. The provisional diagnosis included soft tissue injury, a left ZMC fracture, a left zygomatic arch fracture, a left orbital floor fracture, and a left globe rupture with globe avulsion. Further analysis confirmed a comminuted displaced fracture of the left zygoma, displaced fractures of the left orbit, left orbital floor fracture, comminuted displaced fractures of the maxillary sinus, and rupture of the left eye globe, as shown in Figure 2 and Figure 3.

**Figure 4:**
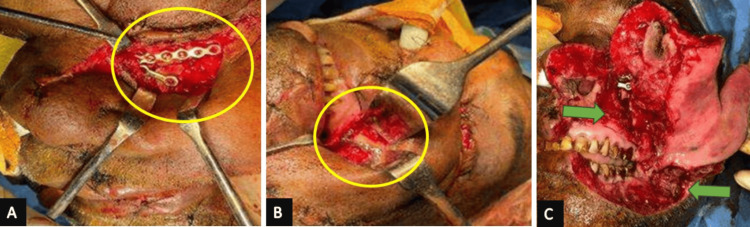
Intraoperative gross view (A) Subtarsal incision given about left side with exposure of the fractured site and plating done using a 1.5-mm (millimeter) eight-hole continuous plate and secured with 1.5x6-mm screws at the left infraorbital margin and 2-mm two-hole plate with gap secured with 2x8-mm screws inferiorly. (B) Stay sutures along the left upper vestibule were removed, dissection was done in layers, and the fracture site was exposed in the left buttress region, which was fixed with a 2-mm four-hole L plate secured with 2x8-mm screws. (C) Stay sutures were removed, the wound was debrided, gentamicin-betamethasone wash was given thoroughly, and closure was done in layers (major lesions of the wound have been marked). The regions of importance have been marked for better understanding

The treatment plan commenced with wound debridement, followed by open reduction and internal fixation under general anesthesia. A sub-tarsal incision on the left side provided access to the fractured site. Plating included a 1.5-mm eight-hole continuous plate at the left infraorbital margin and a 2-mm two-hole plate with a gap in the inferior region. The left orbital floor is reconstructed with prolene mesh secured with a 4.0 vicryl suture. The left buttress region received attention with a 2-mm four-hole L plate. Post-fixation, wound debridement with gentamicin and Betadine wash was conducted, and closure was executed in layers, as shown in Figure 4. Postoperative X-rays confirmed precise implant placement.

**Figure 5:**
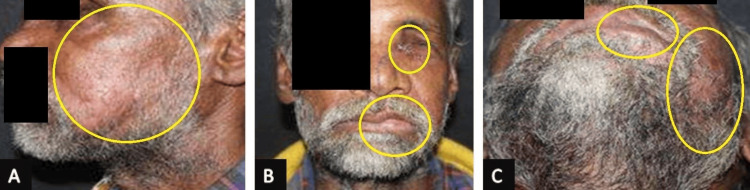
Seven days postoperative picture showing satisfactory healing (A) Left lateral view. (B) Front (anterior) view. (C) Inferior view. The healed/healing regions have been specifically marked which were particularly inspected during follow-up

The patient was discharged two days after the surgery. Medications were prescribed to the patient, such as antibiotics, namely, amoxicillin-clavulanate (Augmentin) 875 mg twice a day and clindamycin 300 mg twice a day (to avoid infection), and nonsteroidal anti-inflammatory drug, namely, ibuprofen 600 mg twice a day (for pain and fever management). The patient was also advised to take a booster vaccination for tetanus toxoid. Follow-up instructions were also given, emphasizing the importance of keeping the surgical site clean and dry, monitoring for signs of infection like pus formation, and attending scheduled follow-up appointments after seven days with the oral and maxillofacial surgeon and an ophthalmologist. The patient exhibited satisfactory healing during the seven-day postoperative follow-up, as shown in Figure 5.

The patient was advised on a tailored rehabilitation plan following his surgery. Initially, he was to manage pain and prevent infection with prescribed antibiotics and analgesics. As his healing progressed, he was advised to fit a custom ocular prosthesis to restore facial aesthetics under the guidance of a prosthodontist. He engaged in physical therapy to maintain facial muscle tone and prevent atrophy. He also underwent visual rehabilitation to adapt to monocular vision, enhancing his depth perception and field adjustment. Psychological support was recommended to help him cope with the psychological impacts of his injury. Regular follow-ups were scheduled to monitor his recovery and adjust the treatment plan as necessary.

## Discussion

Addressing complex hemifacial avulsion injuries presents a significant treatment challenge that demands a multidisciplinary approach. This was a unique case as, according to previous literature, the range of bull gore injuries varies from the abdominal, chest, and scrotal injuries and bull gore injuries to head and neck regions are less common [2,3]. This case of bull gore injury had only facial injury-degloving injury and no injury to other body parts. The first impact of the bull horn was intraorally, which penetrated deep and led to degloving on the left side of the face. Hence, the facial injuries resulting from bull goring are a distinct entity and are not equivalent to injuries caused by other types of trauma seen in regular practice. 

Managing extensive and interconnected facial avulsion and degloving wounds requires a collaborative and multidisciplinary strategy. The coordinated efforts of the interdisciplinary team facilitated specialized planning for primary repair and well-informed decision-making, ultimately resulting in favorable reconstructive outcomes for the patient. This team approach aligns with the principles highlighted by Truong in their study [3]. The significance of early single-stage primary reconstruction was evident in achieving both functional and cosmetic success, in concordance with the observations made by Ghosh and Panse et al. [4,5]. Immediate intervention within injury was pivotal in enhancing the chances of better outcomes, minimizing the risk of infection, and optimizing soft tissue reconstruction.

As explained by Brauner et al. [6], the dental management was taken care of. Hygiene and post-surgical care (like keeping the wounded area hygienic and avoiding going to places with dust to avoid infection) were advised to prevent complications. Eyelid lacerations received specific attention from ophthalmologists within the optimal timeframe hours. The careful repair of lacerations involving the medial canthus, lacrimal, and tarsal glands was conducted meticulously, ensuring positive outcomes through the comprehensive closure of eyelid margins in layers as described by Chang and Rubin [7].

Radiological investigations also aided well during the intraoperative phase and the post-surgical follow-ups, as also evidenced by Ahmed et al. [8]. Moreover, Chhabra et al. [9] also delved into a multidisciplinary approach towards such injuries; hence, ensuring appropriate case management was adopted in this case as well. 

## Conclusions

This case with severe hemifacial avulsion injuries resulting from a bull gore incident underscored the complexities involved in managing high-velocity maxillofacial trauma. This case exemplified the effectiveness of maintaining original tissue viability during reconstruction and the necessity for meticulous postoperative care to prevent complications, thereby setting a precedent for handling similar challenging facial trauma cases. Moreover, the case was more complex and rare, with the trauma to the eyes along with the facial fractures and injuries. Hence, the successful management through immediate, comprehensive single-stage reconstruction highlights the critical role of prompt and effective multidisciplinary intervention. The rehabilitation process and strategy was another facet where the multidisciplinary approach worked wonders. 
